# Epstein-Barr virus microRNAs regulate B cell receptor signal transduction and lytic reactivation

**DOI:** 10.1371/journal.ppat.1007535

**Published:** 2019-01-07

**Authors:** Yan Chen, Devin Fachko, Nikita S. Ivanov, Camille M. Skinner, Rebecca L. Skalsky

**Affiliations:** Vaccine and Gene Therapy Institute, Oregon Health and Science University, Beaverton, Oregon, United States of America; Tulane University School of Medicine, UNITED STATES

## Abstract

MicroRNAs (miRNAs) are post-transcriptional regulatory RNAs that can modulate cell signaling and play key roles in cell state transitions. Epstein-Barr virus (EBV) expresses >40 viral miRNAs that manipulate both viral and cellular gene expression patterns and contribute to reprogramming of the host environment during infection. Here, we identified a subset of EBV miRNAs that desensitize cells to B cell receptor (BCR) stimuli, and attenuate the downstream activation of NF-kappaB or AP1-dependent transcription. Bioinformatics and pathway analysis of Ago PAR-CLIP datasets identified multiple EBV miRNA targets related to BCR signal transduction, including GRB2, SOS1, MALT1, RAC1, and INPP5D, which we validated in reporter assays. BCR signaling is critical for B cell activation, proliferation, and differentiation, and for EBV, is linked to reactivation. In functional assays, we demonstrate that EBV miR-BHRF1-2-5p contributes to the growth of latently infected B cells through GRB2 regulation. We further determined that activities of EBV miR-BHRF1-2-5p, EBV miR-BART2-5p, and a cellular miRNA, miR-17-5p, directly regulate virus reactivation triggered by BCR engagement. Our findings provide mechanistic insight into some of the key miRNA interactions impacting the proliferation of latently infected B cells and importantly, governing the latent to lytic switch.

## Author summary

Understanding the molecular mechanisms regulating EBV latency and entry into the lytic replication cycle is important in developing therapies for viral disease. We demonstrate here that a subset of EBV miRNAs target host factors within the BCR signaling pathway and consequently, negatively regulate cellular responses to BCR cross-linking. Disrupting activity of individual EBV miRNAs, specifically miR-BHRF-1-2-5p and miR-BART2-5p, enhanced lytic gene expression following BCR engagement, suggesting a key role for these miRNAs in attenuating viral reactivation. Our experiments establish a link between EBV miRNAs and signaling through the BCR, providing new insight into miRNA-mediated aspects of latency and reactivation.

## Introduction

Epstein Barr virus (EBV) is a human gamma-herpesvirus that infects >90% of adults worldwide. Primary infection is often asymptomatic or presents as infectious mononucleosis. In immunocompromised individuals, the virus is linked to post-transplant lymphoproliferative disease (PTLD) and hematological malignancies including Burkitt’s (BL) lymphoma, Hodgkin lymphoma, and AIDS-related lymphoma; cancers of epithelial origin such as gastric and nasopharyngeal carcinomas are also associated with EBV infection [1,2]. Following EBV transmission through the oral cavity and subsequent infection of naïve B cells, EBV co-opts multiple aspects of normal B cell activation that induces cell proliferation, initiates differentiation programs, and can drive infected B cells through germinal center (GC) reactions to establish latency in the memory B cell compartment [3–5]. Periodic virus reactivation can occur throughout life-long infection of the host and is thought to help maintain the pool of latently infected cells [5,6]. Like all herpesviruses, EBV has both latent and lytic replication phases, and key to the success of long-term persistence is the ability to navigate between these phases.

EBV encodes over 85 open reading frames (ORFs) and several non-coding RNAs (ncRNAs) which are temporally and differentially expressed throughout the viral life cycle. At least three distinct latency programs (I, II, III) have been described for EBV, distinguished by viral gene expression patterns [7]. The latency III profile consists of nine latent genes (EBNA1, EBNA2, EBNA3A-C, EBNA-LP, LMP2A/B, LMP1) as well as ncRNAs such as the EBERs and 25 precursor microRNAs (pre-miRNAs) which are processed into >40 mature viral miRNAs. Three EBV BHRF1 pre-miRNAs (miR-BHRF1-1, -2, -3), encoded in the BHRF1 locus, are highly expressed following de novo infection, lytic replication, and during latency III when the Cp or Wp promoters are active, such as in lymphoblastoid cell lines (LCLs) derived in vitro [8]. BHRF1 miRNAs can also be detected in some EBV+ B cell tumors [9]. With the exception of miR-BART2, which is encoded anti-sense to EBV BALF5, the remaining EBV miRNAs are clustered within the BART region and are detectable at varying levels in all EBV infection stages (reviewed in [10]).

While exact functions for EBV miRNAs continue to emerge, previous studies demonstrate that these small, viral ncRNAs act akin to their cellular counterparts to post-transcriptionally regulate gene expression via interactions with both viral and cellular RNA targets. EBV and other g-herpesvirus miRNAs are known to regulate multiple cell signaling pathways, including those associated with innate and cell-mediated anti-viral immune responses such cytokine and interferon signaling [11–14]. Specific examples include EBV miR-BHRF1-3, which downregulates the chemokine CXCL11 in primary lymphomas [9]; miR-BART15 can target NLRP3 to alter inflammasome activation [15]; EBV miR-BART16-5p targets CREB-binding protein [16] and can disrupt type I IFN signaling [14]; EBV miR-BHRF1-2-5p targets IL1R1, encoding the major IL-1 receptor, thereby dampening IL-1 signaling [11]. In addition to IL1R1, we recently reported that the EBV BHRF1-2 miRNAs target other IL-1 signaling components such as SOS1, a Ras GDP/GTP exchange factor, and PLCG1, encoding phospholipase C gamma 1 that contributes to receptor-mediated tyrosine kinase signal transduction [11]. Both of these components are integral to many other signaling pathways, such as B cell receptor (BCR) signaling. Furthermore, published miRNA targetome studies suggest that EBV miRNAs target several cellular components related to B cell activation [17–19], leading us to hypothesize that the EBV miRNAs might functionally regulate signal transduction through the BCR.

Aberrant BCR signaling is a hallmark of cancer progression; somatic mutations in cellular components that result in constitutively active or tonic BCR signaling contribute to enhanced proliferation of malignant B cells in cancers such as lymphoma [20,21]. BCR signaling is indispensable for normal B cell activation and differentiation. Engagement of the BCR occurs predominantly through antigen triggers that induce phosphorylation of cytoplasmic CD79a and CD79b immune receptor tyrosine-based activation motifs (ITAMs) by Src family of tyrosine kinases, thereby recruiting signalsome components such as Syk, Btk, Vav guanine exchange factors, and Grb2 and BLNK adaptor proteins to relay signals downstream [22]. Induction of BCR signaling subsequently results in activation of multiple transcription factors, such as NF-kappaB and Jun, which in turn induce genes that participate in B cell proliferation and survival.

In the context of EBV infection, antigenic stimulation of the BCR can initiate plasma cell differentiation, which triggers the switch from latency to lytic replication [5,23,24]. Notably, EBV encodes viral proteins that can provide surrogate BCR survival signals thought to be necessary for establishing and maintaining persistent infection. In latency III cells, EBV-encoded latent membrane protein 2A (LMP2A) recruits Src and Syk tyrosine kinases via an ITAM within its cytoplasmic N-terminal domain, thereby mimicking the BCR [25]. LMP2A has growth transforming properties and may functionally replace BCR signals in B cells lacking an intact BCR [25–27]. Recent proteomic studies provide evidence that additional EBV-encoded factor(s) target the BCR complex for proteasomal degradation during entry into the lytic cycle [28]. While roles for EBV proteins in modulating BCR signal transduction have been described, roles for EBV miRNAs are not yet known.

In this study, we sought to determine whether EBV miRNAs could influence BCR signaling and to subsequently elucidate the molecular mechanism(s) by which this may occur by determining cellular targets involved. To understand how the identified EBV miRNA target interactions might be relevant to the EBV life cycle, we examined gain and loss of function outcomes for specific EBV miRNAs in the growth of latently infected B cells as well as consequences for lytic reactivation triggered through surface Ig cross-linking.

## Results

### Functional screens identify multiple EBV miRNAs that block BCR signaling

**Fig 1:**
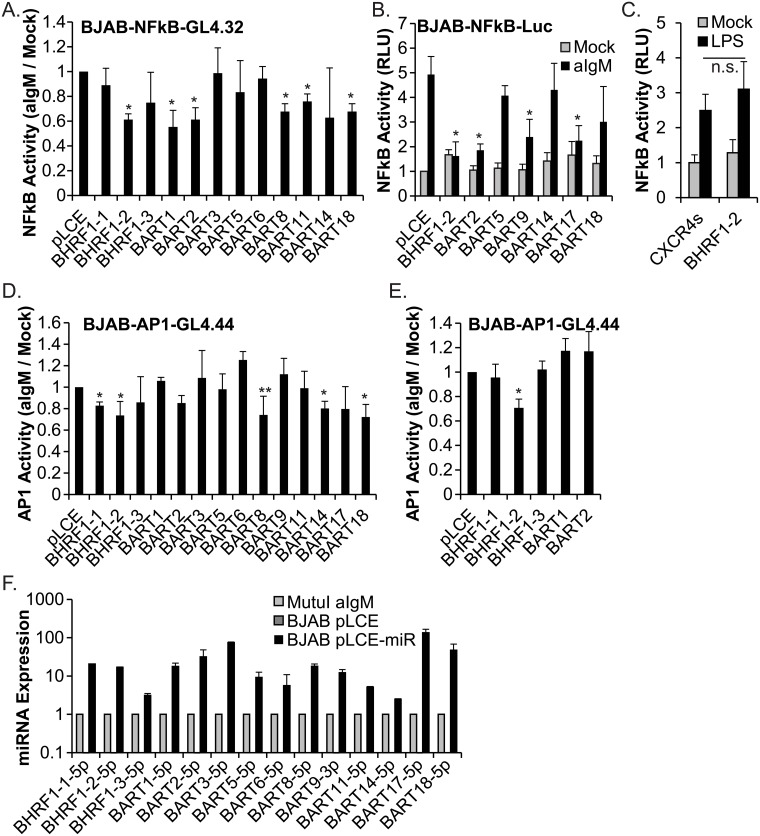
EBV miRNAs disrupt BCR-mediated signaling events. A. BJAB-NFkB-GL4.32, stably transfected with a NF-kappaB-responsive firefly luciferase reporter, were transduced with pLCE empty vector or individual EBV miRNA vectors. Cells were plated in 96-well black-well plates, treated with 10 ug/mL anti-IgM for 18 hrs, and analyzed for luciferase activity. Values are normalized to mock treated cells and reported relative to pLCE control. Shown are the averages of three independent experiments performed in quadruplicate. B. BJAB-NFkBLuc cells, expressing a NF-kappaB-responsive firefly luciferase reporter and a renilla luciferase reporter for internal control, were transduced with pLCE empty vector or individual EBV miRNA vectors. Cells were treated with 5 ug/mL anti-IgM for 18 hr, and then lysed in 1X passive lysis buffer. Luciferase activity was measured using the dual luciferase reporter kit. Values are reported relative to mock-treated control (pLCE) cells. Shown are the averages of four independent experiments performed in triplicate. C. BJAB-NFkBLuc cells transduced with pLCE or pLCE-BHRF1-2 were stimulated with 100 ng/mL LPS for 6 hrs, then analyzed for luciferase activity as in (A.). Shown are the averages of three independent experiments performed in triplicate. D. and E. BJAB-AP1-GL4.44 cells, expressing an AP1-responsive firefly luciferase reporter, were transduced with pLCE-based EBV miRNA expression vectors, treated with 10 ug/mL anti-IgM for 6 hrs (D.) or 18 hrs (E.), then analyzed for luciferase activity. Values are normalized to mock treated cells. Shown are the averages of three independent experiments performed in quadruplicate. F. EBV miRNA expression. RNA was harvested from BJAB cells transduced with control vector (pLCE) or individual EBV miRNA expression vectors (pLCE-miR) and Mutu I cells treated with 5 ug/mL anti-IgM for 48 hrs. miRNAs were detected by qRT-PCR. Values are normalized to cellular miR-16 and reported relative to levels in Mutu I cells. By Student’s t-test, *p<0.05 or **p = 0.054. RLU = relative light units.

To determine if EBV miRNAs could functionally regulate signaling through the BCR, we screened individual viral miRNAs in EBV-negative BJAB BL cells which express surface IgM and are responsive to antigenic triggering of the BCR using antibodies against IgM (Fig 1). BJAB cells stably expressing a NF-kappaB luciferase reporter (BJAB-NFkB-GL4.32) were transduced with EBV miRNA expression vectors and then, cells were treated with anti-IgM to ligate the BCR and activate downstream signaling. We initially tested all three BHRF1 miRNAs, miR-BART2, four BART miRNAs from BART Cluster 1 (miR-BART1, 3, 5, and 6) and four BART miRNAs from Cluster 2 (miR-BART8, 11, 14, and 18) (Fig 1A). Notably, expression of six miRNAs (BHRF1-2, BART1, BART2, BART8, BART11, and BART18) significantly impacted the amplitude of the response to BCR stimulation as measured by reduced NF-kappaB activity. To examine whether basal NF-kappaB activity might be affected by EBV miRNA expression, we also tested several miRNAs in BJAB-NFkB-Luc cells [11] that constitutively express an internal control renilla luciferase in addition to a firefly luciferase NFkB reporter (Fig 1B). We included BART9 and BART17 in these subsequent experiments based on published miRNA targetome studies indicating these two miRNAs might target the BCR signaling pathways [17, 18]. In general, basal NFkB activity was not affected, while addition of anti-IgM led to a ~5-fold increase in NFkB reporter activity in pLCE control cells (Fig 1B). Consistent with our initial experiments, BHRF1-2 and BART2 miRNAs reproducibly attenuated BCR signaling responses; we additionally observed decreased NFkB activity in the presence of BART9 and BART17 miRNAs (Fig 1B). Together, these experiments identified eight EBV miRNAs that significantly reduced NFkB responses initiated through the BCR.

We have recently demonstrated that the EBV BHRF1-2 miRNAs can reduce NF-kappaB activation in response to IL-1 cytokines [11]. There is extensive cross-talk between cytokine, BCR, and toll-like receptor (TLR) signaling pathways, leading us to consider the possibility that the BHRF1-2 miRNAs may be inhibiting core NF-kappaB components or other factors common amongst these pathways. We therefore examined the TLR4 signaling response by treating cells with lipopolysaccharide (LPS). Compared to control cells, the presence of BHRF1-2 miRNAs had no impact on LPS-mediated NF-kappaB activation (Fig 1C). These results indicate that activity of the BHRF1-2 miRNAs is directed more towards IL-1 and BCR pathways.

Engagement of the BCR also activates JNK (c-Jun N-terminal kinases) and p38, triggering MAPK signaling, and regulating activity of AP1 (Activator protein 1) family transcription factors such as JUN and ATF2 [29,30]. Previously, miR-BART18-5p was shown to target the MAP3K2 (MAP kinase kinase kinase 2) 3’UTR, suggesting that downstream transcription mediated through ATF2 and c-Jun may be affected [31]. Thus, to determine if AP1-dependent transcription is indeed influenced by EBV miRNAs, we generated BJAB cells stably expressing an AP1 luciferase reporter (BJAB-AP1-Luc) and measured responses to BCR cross-linking following 6 hr anti-IgM stimulation. Expression of the BHRF1-1, BHRF1-2, BART14, or BART18 miRNAs significantly attenuated AP1 activation (Fig 1D). We also tested 18 hr anti-IgM stimulation and observed significantly reduced AP1 activity in the presence of the BHRF1-2 miRNAs (Fig 1E), indicating these miRNAs are highly involved in controlling BCR responses. Expression levels for individual EBV miRNAs in transduced BJAB cells are shown in Fig 1F. Together with the NF-kappaB reporter assays, these experiments demonstrate that multiple EBV miRNAs (including BHRF1-2, BART2, BART9, BART17, and BART18) can functionally attenuate signaling events initiated through BCR engagement. As BJAB cells are not infected with EBV, these results further show that desensitization to BCR stimuli occurs through viral miRNA actions on the host cell, presumably through the partial inhibition or complete silencing of cellular targets.

### Cellular mRNA targets of EBV miRNAs are associated with BCR signaling

**Fig 2:**
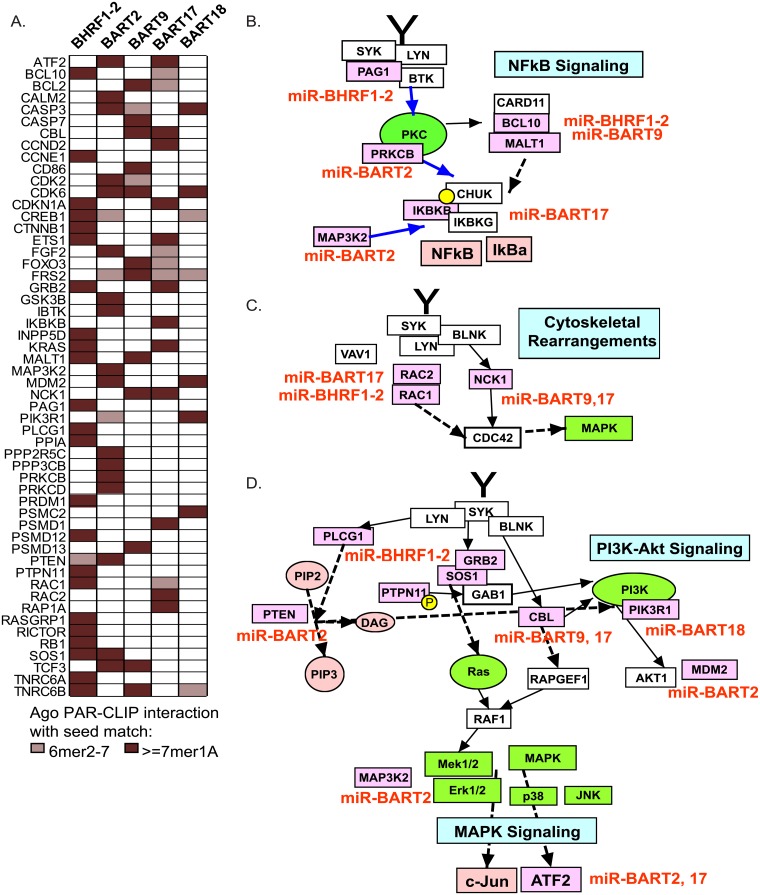
Cellular targets of EBV miRNAs involved in BCR signaling pathways. A. EBV miRNA interactions identified in PAR-CLIP datasets from EBV B95-8 or wild-type LCLs and EBV+/KSHV+ BC1 cells. CLIP’ed cellular 3’UTR sites with > = 6mer canonical seed matches (nt 2–7) to the indicated EBV miRNAs were compared to a BCR-associated gene list curated from six databases (see Methods). Red boxes indicate presence of an interaction site for a specific 3’UTR. B.-D. Pathways downstream of the BCR are targeted by EBV miRNAs. Cellular targets are highlighted in pink with the corresponding EBV miRNA(s) listed in red. Pathways were constructed in PathVisio 3.0.

In order to identify the molecular mechanisms by which EBV miRNAs attenuate BCR signaling, we queried published B cell Ago PAR-CLIP datasets for experimentally derived, protein-coding mRNA targets of EBV miRNAs, focusing specifically on cellular targets for EBV BART2, BART9, BART17, BART18, and BHRF1-2 miRNAs. Combined datasets included EBV B95-8 LCLs (expressing BHRF1-2 and BART2 miRNAs, among others), two EBV wild-type LCLs (expressing all EBV miRNAs), and one EBV+/KSHV+ PEL (expressing all 22 BART miRNAs) [17,18,32]. 3,501 target 3’UTR interactions (representing 1,891 unique human genes) with 6mer (nt 2–7) or greater seed match sites were extracted for the five EBV miRNAs and subsequently, analyzed for genes with established roles in BCR signaling using a manually curated list from six combined public resource pathway collection databases (Reactome, Panther Pathways, NDeX, DAVID 6.8 (KEGG and Biocarta), PathCards, and NetPath) [33–38]. Based upon the literature, we also included INPP5D (encodes the co-stimulatory phosphatase SHIP1), PRDM1 (encodes BLIMP1, a master regulator of B cell differentiation), and PAG1 (encodes a phosphoprotein that associates with Lyn and/or Fyn [39]). A total of 54 EBV miRNA targets were associated with BCR signaling including genes directly involved in Signaling by the B cell Receptor (Reactome pathway R-HSA-983705) and involved in B cell activation (Panther pathway P00010) (Fig 2A). Targets include the SH2-domain containing adaptor Growth factor receptor-bound protein 2 (GRB2) and Grb2 binding partners (SOS1) that coordinate signaling downstream of the BCR to facilitate activation of Ras, MAPK, PI3K, and indirectly, NF-kappaB [40,41] as well as the RAC1 (Ras-related C3 botulinum toxin substrate 1) GTPase that is involved in cytoskeletal dynamics and critically required for B cell development [42] (Fig 2C and 2D). Additional noteworthy targets include a core NF-kappaB signaling component Ikk-beta (IKBKB) and members of the Mucosa-associated lymphoid tissue lymphoma translocation protein 1 (MALT1) signaling complex (MALT1, BCL10) that modulate NF-kappaB activation (Fig 2B).

### Luciferase 3’UTR reporter assays confirm EBV miRNA target interactions

**Fig 3:**
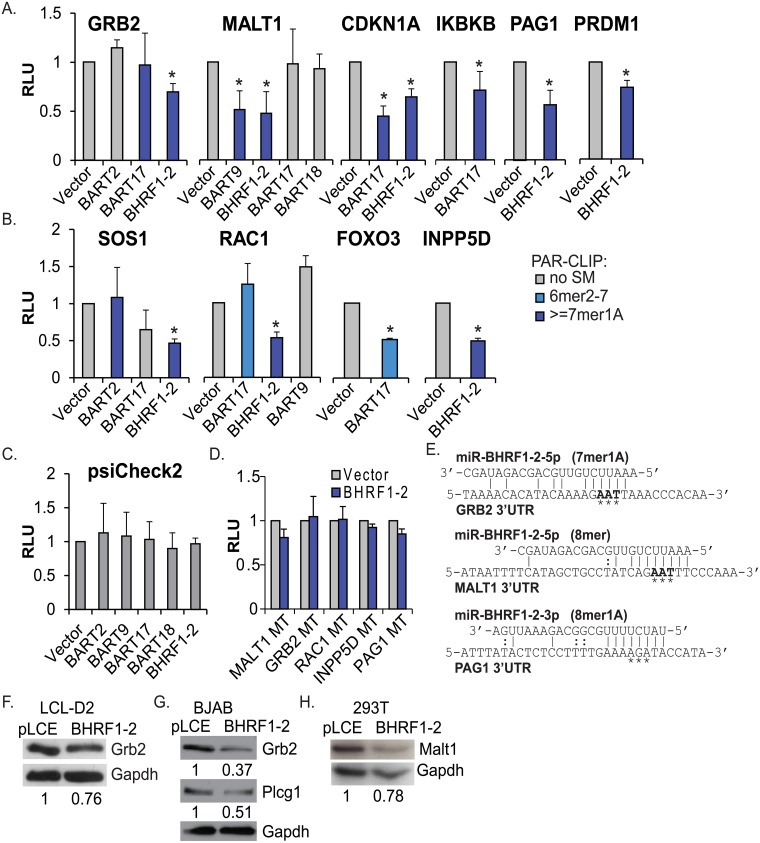
Validation of EBV miRNA targets. A-C. 293T cells were co-transfected with indicated 3’UTR luciferase reporters or psiCheck2 empty vector (C.) and EBV miRNA expression vectors (pLCE-based). 48–72 hrs post-transfection, cells were lysed and assayed for dual luciferase activity. PAR-CLIP interactions between EBV miRNAs and 3’UTRs are highlighted. SM = seed match. Reported are the averages of at least three independent experiments performed in triplicate. *By Student’s t-test, p<0.05. RLU = relative light units. D. Site-directed mutagenesis of BHRF1-2 miRNA seed match sites in target 3’UTRs abrogates knockdown. Luciferase assays were performed as described in A-C. Reported are the averages of at least three independent experiments performed in triplicate. E. Alignments showing the BHRF1-2 miRNA interaction sites and mutations (starred nucleotides) generated for the GRB2, MALT1, and PAG1 3’UTR reporters. F-H. Grb2, Plcg1, and Malt1 are inhibited in the presence of EBV miRNAs. In F. and G., western blot analysis was performed on lysates from BHRF1-2 miRNA mutant LCLs (LCL-D2) or EBV negative BJAB cells ectopically expressing GFP (pLCE) or the BHRF1-2 miRNAs. In H., western blot analysis was performed on lysates from 293T cells transiently transfected with pLCE-based miRNA expression vectors (72 hr post-transfection). Gapdh levels are shown as loading controls. Band intensities were quantified using ImageJ, normalized to loading controls, and reported relative to control cells (WT or pLCE).

From the list of target genes related to BCR signaling, we selected several 3’UTRs for further investigation by conventional 3’UTR reporter assays; this included the GRB2 and MALT1 3’UTRs which are potentially regulated by multiple EBV miRNAs (Fig 2A). With the exceptions of the CDKN1A and PRDM1 3’UTRs which are expressed from pLSG [18], 3’UTRs are expressed from the dual luciferase reporter vector, psiCheck2. Reporters were co-transfected with individual EBV miRNA expression vectors into 293T cells, lysates were collected 48–72 hrs post-transfection, and luciferase activity was measured. Luciferase knockdown was observed for 12 out of 15 PAR-CLIP-identified miRNA interactions, confirming these cellular 3’UTRs as targets of the EBV miRNAs (Fig 3A–3C).

To further investigate 3’UTR interactions for the BHRF1-2 miRNAs, we then used site-directed mutagenesis to disrupt individual seed-match sites, focusing our efforts on the MALT1, GRB2, RAC1, INPP5D, and PAG1 3’UTRs. The Ago-CLIP identified interaction between miR-BHRF1-2-3p and the PRDM1 3’UTR was recently confirmed [17,43]. Disrupting the seed match sites fully alleviated miRNA-mediated luciferase knockdown in all instances (Fig 3D) thereby demonstrating that these are indeed bona fide interaction sites for the EBV BHRF1-2 miRNAs, and further specifying direct targeting by either the 3p or 5p miRNA (both miRNAs are generated by the BHRF1-2 vector). Fig 3E shows examples of the mutations made for three of the 3’UTRs tested. Inhibition of the MALT1 3’UTR by EBV miR-BHRF1-2-5p in luciferase assays was recently reported [44]; here, our experiments confirm the PAR-CLIP studies that mapped the miRNA interaction site to a single site within the first 800 nt of the MALT1 3’UTR downstream of the stop-codon (Fig 3E) [17]. In total, we validated eight targets for the EBV BHRF1-2 miRNAs (GRB2, MALT1, CDKN1A, PAG1, INPP5D, RAC1, PRDM1, SOS1) that are associated with BCR signaling and further demonstrate through these assays that the MALT1 and CDKN1A 3’UTRs can be regulated by more than one EBV miRNA.

### Host targets are responsive to EBV miRNA activity at the protein level

To ask if the targets of EBV miRNAs validated above exhibited changes at the protein level, we first examined Grb2 protein levels in BHRF1-2 miRNA mutant LCLs or BJAB cells that were stably transduced with pLCE control vector or pLCE-BHRF1-2 to express the BHRF1-2 miRNAs in trans [11]. Consistent with inhibition of the GRB2 3’UTR luciferase reporter, we observed a ~25% decrease in Grb2 levels in LCLs and >70% reduction in Grb2 in BJAB cells upon introduction of the BHRF1-2 miRNAs (Fig 3F and 3G). In BJAB cells, we also observed reduced phospholipase C gamma 1 (Plcg1) levels, which we have recently demonstrated is regulated by miR-BHRF1-2-3p [11,17]. To test other targets, miRNA expression vectors were transfected into 293T cells and lysates were analyzed for protein expression. By Western blot assays, we observed a decrease in Malt1 levels (Fig 3H) in the presence of the BHRF1-2 miRNAs. These data, together with the luciferase reporter assays, thus provide experimental evidence for multiple targets of the EBV miRNAs that are commonly linked to BCR signaling pathways.

To examine targets in the context of EBV infection, we also compared Grb2 and Malt1 protein levels in established, donor-matched EBV B95-8 wild-type LCLs versus BHRF1-2 miRNA mutant LCLs that lack both miR-BHRF1-2-3p and miR-BHRF1-2-5p [11,45]. Malt1 was upregulated in BHRF1-2 miRNA mutant LCLs for two out of three donor pairs and Grb2 was upregulated in BHRF1-2 miRNA mutant LCLs for one of the two donor pairs tested (S1A and S1B Fig), demonstrating that loss of BHRF1-2 miRNA activity in latently infected cells can confer enhanced protein expression. We further assessed target RNAs by qRT-PCR in EBV B95-8 wild-type and BHRF1-2 miRNA mutant LCLs. For most targets, steady state levels were not reproducibly affected by the presence or absence of the BHRF1-2 miRNAs (S1C–S1G Fig), suggesting that mRNA repression by the BHRF1-2 miRNAs may not generally lead to mRNA degradation. Alternatively, mRNA stability for these BHRF1-2 targets may be impacted during a specific stage of EBV infection that is not captured in established LCLs. We did, however, observe significant knockdown of GRB2 transcripts upon ectopic expression of the BHRF1-2 miRNAs in BJAB cells, demonstrating that GRB2 mRNAs may be subject to BHRF1-2 miRNA mediated degradation or decay under certain conditions (S1H Fig).

### shRNA inhibition of RAC1, GRB2, or SOS1 mimics EBV miRNA activity in NF-kappaB reporter assays

**Fig 4:**
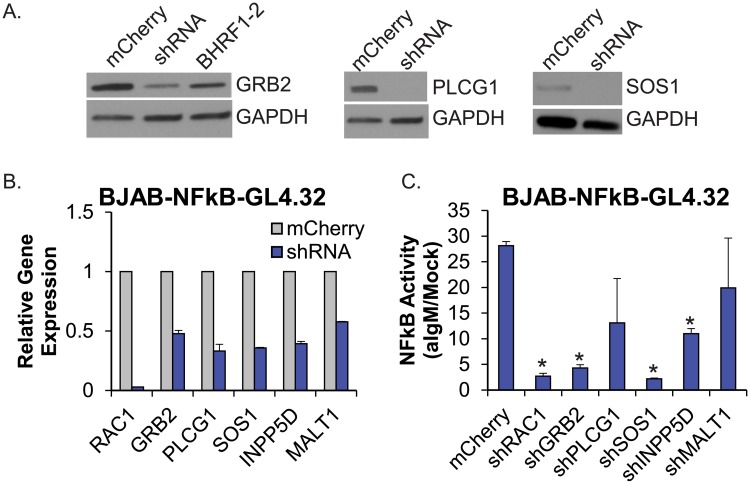
shRNAs to RAC1, GRB2, or SOS1 phenocopy EBV BHRF1-2 miRNA activity. A. BJAB cells were transduced with pL-mCherry or shRNAs to GRB2, PLCG1, or SOS1 as indicated. Lysates were analyzed by Western blot. Gapdh levels are shown as loading controls. Lysate from BJAB cells transduced with pLCE-BHRF1-2 was included in the Grb2 Western blot. B. Knockdown of individual target genes in BJAB-NFkB-GL4.32 cells was assayed by qRT-PCR analysis. Expression levels are normalized to GAPDH and reported relative to control (mCherry) cells. qPCR was performed in duplicate. C. Individual shRNAs were stably expressed in BJAB-NFkB-GL4.32 cells. Cells were stimulated for 18 hr with 5 ug/ml anti-IgM, then lysed and assayed for NF-kappaB responsive luciferase activity. NF-kappaB activity levels are normalized to mock treated cells. Averages and standard deviations (S.D.) are from two independent experiments performed in quadruplicate. By Student’s t-test, *p<0.05.

Having identified several BCR signaling components as EBV miRNA targets, we then asked whether RNAi-mediated knockdown of the individual target RNAs could potentially phenocopy EBV miRNA function. We generated shRNAs (short hairpin RNAs) for individual cellular targets of the EBV BHRF1-2 miRNAs since activity of both NF-kappaB and AP1 reporters was reduced by these miRNAs in response to anti-IgM stimulation (Fig 1). miR-30-based shRNAs generated against RAC1, GRB2, PLCG1, SOS1, INPP5D, and MALT1 were introduced into BJAB cells; qRT-PCR and/or Western blot analysis was performed to confirm that each shRNA construct appropriately knocked down their target of interest (Fig 4A and S2A Fig). All shRNAs were then introduced into BJAB-NFkB-Luc reporter cells, lentiviral transduction was confirmed by mCherry fluorescence, and transcript levels were assayed by qRT-PCR to confirm knockdown (Fig 4B). Cells were subsequently stimulated with anti-IgM to activate the NF-kappaB reporter. shMALT1, shINPP5D, and shPLCG1 cells remained partially responsive to BCR cross-linking, while shRNA depletion of GRB2, SOS1, or RAC1 potently inhibited NF-kappaB activation (Fig 4C). Notably, GRB2, SOS1, and RAC1 are all targets of miR-BHRF1-2-5p (Fig 3), suggesting that this miRNA, in particular, acts as a key regulator of BCR signal transduction.

### Regulation of GRB2 by miR-BHRF1-2-5p contributes to LCL growth

Previous studies show that genetic ablation of the EBV BHRF1 miRNAs from the viral genome impairs but does not fully inhibit LCL outgrowth in vitro [45–48]. Furthermore, LCLs established with BHRF1 miRNA mutant viruses exhibit altered cell cycle progression and reduced growth rates [46,47]. While these phenotypes can be attributed, in part, to inefficient processing of the BHRF1 RNAs and/or deregulated splicing of EBNA-LP transcripts that occurs in *cis* [49,50], the contributions from specific cellular targets of the BHRF1 miRNAs have not been fully examined. GRB2 encodes a ubiquitously expressed signaling adaptor protein that is recruited to growth factor receptors via its SH2 and SH3 domains, coordinates with SOS1 to recruit Ras GTPases, and subsequently, transduces external signals that can activate genes involved in cell proliferation [40,41]. We therefore hypothesized that targets of the BHRF1-2 miRNAs, such as GRB2 or SOS1, might play a role in the survival and/or proliferation of latently infected B cells.

**Fig 5:**
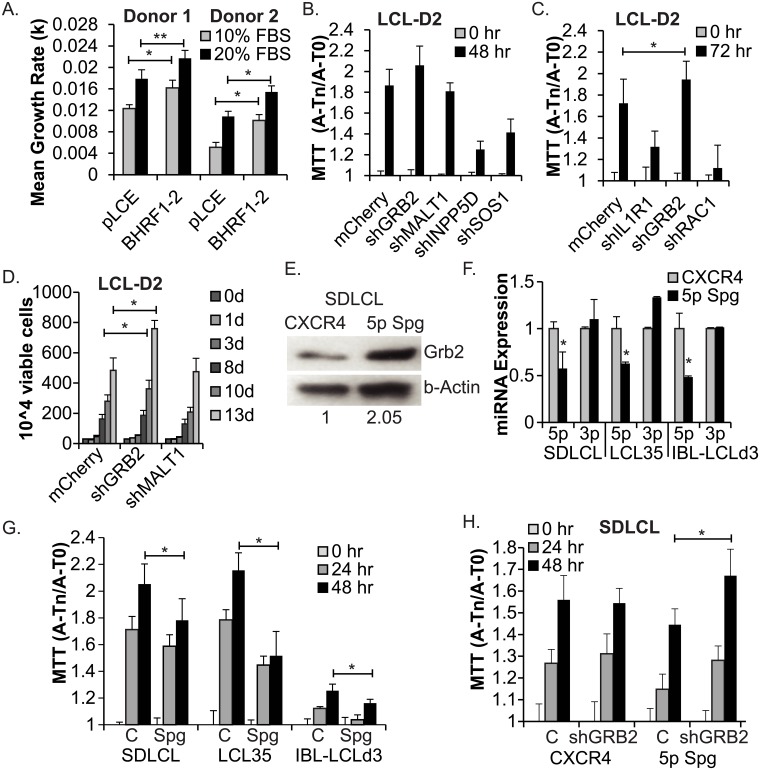
Regulation of GRB2 by EBV miR-BHRF1-2-5p contributes to the growth of latently infected LCLs. A. Ectopic expression of the BHRF1-2 miRNAs enhances the growth of mutant LCLs. BHRF1-2 miRNA mutant LCLs stably transduced with pLCE or pLCE-BHRF1-2 (Donor 1 = LCLBACD2; Donor 2 = LCL-D2) were plated in triplicate or quadruplicate at 2.5 x 10^6 cells per mL in media containing 10% FBS or 20% FBS (see also S3 Fig). Viable cell counts were determined at times indicated in S3 Fig. Cell growth rates were calculated between 2 and 5 days post-plating using the equation: ln(N1/N1) = k(t1-t2), where k = growth rate, t = time, and N = cell number. *By Student’s t-test, p<0.02. B. and C. shRNA knockdown of GRB2 enhances the growth of BHRF1-2 miRNA mutant LCLs. BHRF1-2 miRNA mutant LCLs (LCL-D2) were transduced with pL-mCherry-based shRNAs; cell proliferation was determined by MTT assay. A = Absorbance at 562 nm, T = time, n = 48 or 72 hr as indicated. Values at Tn are normalized to the absorbance values at 0 hr (A-T0). Error bars represent S.D. of measurements from eight wells. D. LCL-D2 cells transduced with pL-mCherry or shRNAs to GRB2 or MALT1 were plated in quadruplicate at 2.5 x 10^6 cells per mL in media containing 20% FBS. Viable cells were counted at days indicated. E. Grb2 protein levels increase in the absence of miR-BHRF1-2-5p activity. Lysate was harvested from EBV B95-8 SDLCL cells transduced with pLCE-CXCR4 control or miR-BHRF1-2-5p sponge inhibitor (5p Spg) 10 dpi and analyzed by Western blot for Grb2 and b-Actin. Band intensities were quantified using Image J. F. Taqman qRT-PCR analysis of miR-BHRF1-2-5p (5p) and miR-BHRF1-2-3p (3p) expression in miR-BHRF1-2-5p sponged LCLs. Values are normalized to U6 and reported relative to the BHRF1-2 miRNA levels in each respective LCL transduced with pLCE-CXCR4 control vector. G. Disruption of miR-BHRF1-2-5p activity reduces LCL growth. Proliferation of EBV B95-8 (SDLCL, LCL35) and wild-type LCLs (IBL-LCLd3) as determined by MTT assay following stable transduction with pLCE-CXCR4 control vector “C” or the miR-BHRF1-2-5p sponge “Spg”. LCLs were maintained in media containing 10% FBS and split one day prior to MTT assays. Values at Tn are normalized to the absorbance values at 0 hr (A-T0). Error bars represent S.D. of measurements from eight wells. *By Student’s t test, p<0.05. H. shRNA knockdown of GRB2 restores LCL growth in the absence of miR-BHRF1-2-5p activity. Cell proliferation was determined by MTT assay as in (G.). mCherry “C” or GRB2 shRNA “shGRB2” were introduced into SDLCL cells stably expressing GFP (CXCR4) or the miR-BHRF1-2-5p sponge. Error bars represent S.D. of measurements from 12 wells. *By Student’s t test, p<0.05.

Using cell viability assays (trypan-blue exclusion) as well as MTT (3-(4,5-dimethylthiazol-2-yl)-2,5-diphenyltetrazolium bromide) assays to monitor metabolic activity, we first measured the growth of established, donor-matched LCLs (>6–8 weeks post-infection) that are latently infected with either EBV B95-8 2089 wild-type virus or a miRNA mutant virus in which both BHRF1-2 miRNAs are deleted [45]. In agreement with prior studies [45,46], LCLs established with the BHRF1-2 miRNA mutant virus grew significantly slower than wild-type LCLs (S3A–S3C Fig). We subsequently asked whether this growth phenotype could be rescued by re-introducing the BHRF1-2 miRNAs. We measured the growth rates of two BHRF1-2 miRNA mutant LCLs which stably express BHRF1-2 miRNAs in trans [11]. Compared to control cells transduced with pLCE empty vector, ectopic BHRF1-2 miRNA expression enhanced LCL growth rates by 20–25% (Fig 5A and S3D and S3E Fig). In an effort to improve the differences in cell proliferation observed between control and BHRF1-2 miRNA expressing cells, we also tested growth rates in 20% serum conditions. As anticipated, LCLs exhibited increased growth rates at higher serum concentrations; however, the measured effect of the BHRF1-2 miRNAs was comparable to the 10% serum conditions, enhancing cellular growth rates by ~20% (Fig 5A). BHRF1-2 miRNA expression in EBV negative BJAB cells had no significant effect on cell growth (S3F Fig), suggesting this phenotype may be specific to the B cell stage of LCLs. These results show that the growth defect conferred through genetic loss of BHRF1-2 miRNAs from the viral genome can be at least partially reversed by complementing mutant LCLs with the BHRF1-2 miRNAs in trans.

To examine whether cellular factors identified above might contribute to LCL proliferation, we then introduced shRNAs into BHRF1-2 miRNA mutant LCLs (LCL-D2) against GRB2, MALT1, INPP5D, SOS1, RAC1, or IL1R1 (regulated by miR-BHRF1-2-5p [11]). Following shRNA transduction, MTT and cell viability assays were performed to monitor changes in growth patterns (Fig 5B–5D). Knockdown of each target transcript was confirmed by qRT-PCR analysis (S2B Fig). MALT1 inhibition had no observable effects on LCL growth, despite achieving ~50% knockdown with this shRNA (S2B Fig). Strikingly, shRNA inhibition of INPP5D, SOS1, IL1R1, or RAC1 in LCL-D2 was detrimental to cell growth, while in three separate experiments, shRNA-mediated reduction of GRB2 lead to a small, but statistically significant increase in LCL-D2 growth (Fig 5B–5D). These results demonstrate dependencies on INPP5D, SOS1, IL1R1, and RAC1 for efficient LCL proliferation and furthermore, indicate that RNAi-mediated control of GRB2 confers a modest growth advantage to latently infected B cells.

The GRB2 3’UTR is targeted by miR-BHRF1-2-5p in luciferase reporter assays (Fig 3). To confirm that this miRNA specifically controls Grb2 expression during EBV infection, we performed Western blot assays. Grb2 protein levels were increased by >2-fold in LCLs transduced with a sponge inhibitor for miR-BHRF1-2-5p (Fig 5E). To examine the relationship between miR-BHRF1-2-5p and LCL growth, we then introduced the miR-BHRF1-2-5p inhibitor into additional LCLs infected with either EBV B95-8 or a wild-type EBV. miRNA knockdown was confirmed by Taqman qRT-PCR and importantly, levels of miR-BHRF1-2-3p were unaffected (Fig 5F). MTT assays were subsequently performed to monitor metabolic activity of miRNA-sponged LCLs. Loss of miR-BHRF1-2-5p function did not critically impair LCL growth, such has previously been observed for miR-155 loss [51]; however, inhibition of the miRNA did significantly attenuate the growth of all three LCLs (Fig 5G). In separate experiments, we monitored viable cell counts for miRNA-sponged LCLs and controls (pLCE-CXCR4s or pLCE empty vector) generated in parallel (S4A–S4C Fig). Congruent with MTT assay results, inhibition of miR-BHRF1-2-5p activity reproducibly reduced cell growth rates by 20–30% (S4A–S4C, S4H and S4I Fig), demonstrating that latently infected B cells are dependent on miR-BHRF1-2-5p function for efficient proliferation.

To ask if we could rescue the growth phenotype conferred by miR-BHRF1-2-5p knockdown, GRB2 shRNAs were expressed in combination with the miR-BHRF1-2-5p sponge and MTT assays were performed (Fig 5G). Consistent with the phenotype observed in BHRF1-2 miRNA mutant LCLs (Fig 5B), shRNA-mediated inhibition of GRB2 reversed the miRNA inhibitor effects and led to increased proliferation of miR-BHRF1-2-5p-sponged LCLs compared to control cells (Fig 5G and S4D–S4G Fig). The increase in cell proliferation was specific to GRB2 since shRNAs against RAC1 or SOS1 did not have a measurable impact on LCL growth (S4D and S4E Fig). Together, these results demonstrate that EBV miR-BHRF1-2-5p plays an active role in promoting the growth of latently infected B cells through the regulation of GRB2.

### Inhibition of miR-BHRF1-2-5p impairs the growth of EBV+ DLBCLs

**Fig 6:**
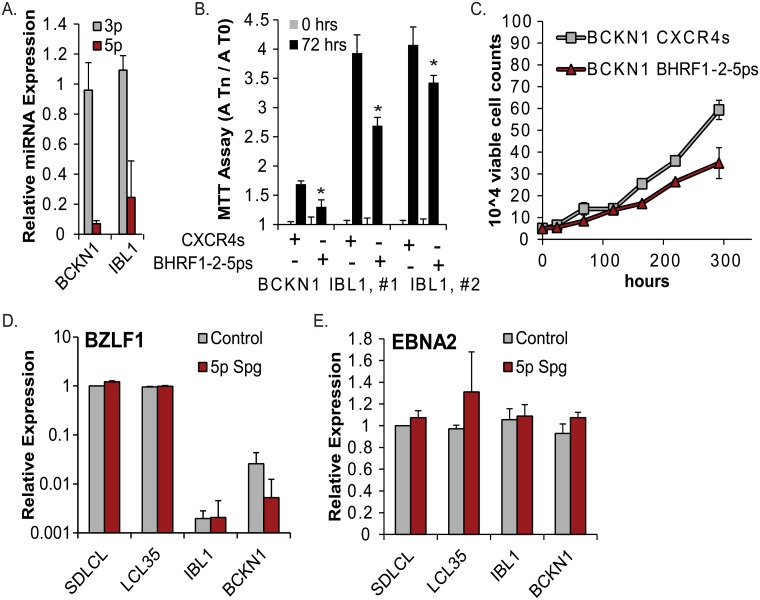
EBV miR-BHRF1-2-5p contributes to the growth of EBV+ DLBCL cells. A. Taqman qRT-PCR analysis of miR-BHRF1-2-5p (5p) and miR-BHRF1-2-3p (3p) expression in miR-BHRF1-2-5p sponged DLBCLs (IBL1 and BCKN1). Values are normalized to U6 and reported relative to the BHRF1-2 miRNA levels in each respective DLBCL transduced with pLCE-CXCR4 control vector. B. Proliferation of DLBCLs as determined by MTT assay following stable transduction with pLCE-CXCR4 control vector or the miR-BHRF1-2-5p sponge. FAC-sorted, GFP+ DLBCLs were maintained in media containing 15% FBS and split one day prior to MTT assays. Values at Tn are normalized to the absorbance values at 0 hr (A-T0). Error bars represent S.D. of measurements from six or eight wells. *By Student’s t test, p<0.05. C. Growth curves of BCKN1 cells expressing the miR-BHRF1-2-5p inhibitor. DLBCLs, at 7 days post-FACS, were plated in media containing 15% FBS. D. and E. Loss of miR-BHRF1-2-5p activity in LCLs or DLBCLs does not induce spontaneous reactivation. BZLF1 and EBNA2 expression levels were assayed by qRT-PCR in two EBV B95-8 LCLs (LCL35 and SDLCL) and two DLBCL lines (IBL1 and BCKN1) transduced with either pLCE control or the miR-BHRF1-2-5p sponge inhibitor. Values are normalized to GAPDH and shown relative to control SDLCL cells. Reported are the averages of two independent experiments; PCR was performed in duplicate.

As LCLs are artificially generated *in vitro*, we next sought to determine whether miR-BHRF1-2-5p also plays a role in maintaining the growth of EBV transformed B cells that are naturally derived. Diffuse large B cell lymphoma (DLBCL) is the most common form of NHL in HIV-infected patients, and >85% of immunoblastic, AIDS-related DLBCL are EBV+ [52]. Using qRT-PCR, we first characterized the viral gene expression pattern in EBV+ DLBCL cell lines [53]. Consistent with a latency III program, both the LMP1 and EBNA2 transcripts were present, as well as high levels of the BHRF1 miRNAs (S5 Fig). We then transduced IBL1 and BCKN1 cells with the miR-BHRF1-2-5p sponge inhibitor. Transduced DLBCL cells expressing high GFP were FAC-sorted, and miRNA knockdown was confirmed by qRT-PCR (Fig 6A). Cell proliferation was subsequently measured by MTT assay (Fig 6B). Similar to LCLs, sponge inhibition of miR-BHRF1-2-5p in the DLBCL cell lines attenuated cell proliferation (Fig 6B). To corroborate these results, we also performed growth curves with FAC-sorted BCKN1 cells, and observed that loss of miR-BHRF1-2-5p activity reduced cell growth by ~20% compared to control cells (Fig 6C). To examine the possibility that spontaneous lytic reactivation was occurring in response to miR-BHRF1-2-5p inhibition and contributing to the decrease in cell proliferation, we monitored viral gene expression in both DLBCLs and LCLs. Neither BZLF1 nor EBNA2 levels were altered in the sponged cells (Fig 6D and 6E), ruling out virus reactivation as a contributing factor in the regulation of B cell proliferation by EBV miR-BHRF1-2-5p.

### Inhibition of miR-BHRF1-2-5p, miR-BART2-5p, or host miR-17-5p enhances BCR-mediated EBV reactivation

Cross-linking of surface Ig on B cells latently infected with EBV can facilitate lytic reactivation [54]. To investigate miRNA activities in EBV+ B cells that are sensitive to BCR stimuli, we first examined miRNA expression in Mutu I BL cells which, like BJAB cells, express membrane-bound IgM on the surface but are latently infected with EBV type 1 and characteristically exhibit a restricted latency type I pattern of viral gene expression (i.e. EBNA1, low levels of BART miRNAs, nearly undetectable levels of BHRF1 miRNAs) [8,55]. Treatment of Mutu I cells with anti-IgM resulted in ~25–35 fold increases in EBV immediate-early, early, and late gene products (BZLF1, BRLF1, BALF4, BNLF2a, and BHRF1) as detected by qRT-PCR (S6A Fig), demonstrating entry into the lytic phase. Consistent with previous reports, we observed rapid induction of miR-BHRF1-2-5p, which correlated with BHRF1 mRNA levels, and a delay in the induction of miR-BHRF1-1-5p following lytic reactivation [8,56] (S6B Fig). Notably, all EBV BART miRNAs that functionally inhibited BCR signaling (miR-BART2-5p, miR-BART9, miR-BART17, and miR-BART18) were upregulated during lytic reactivation (S6B and S6C Fig).

**Fig 7:**
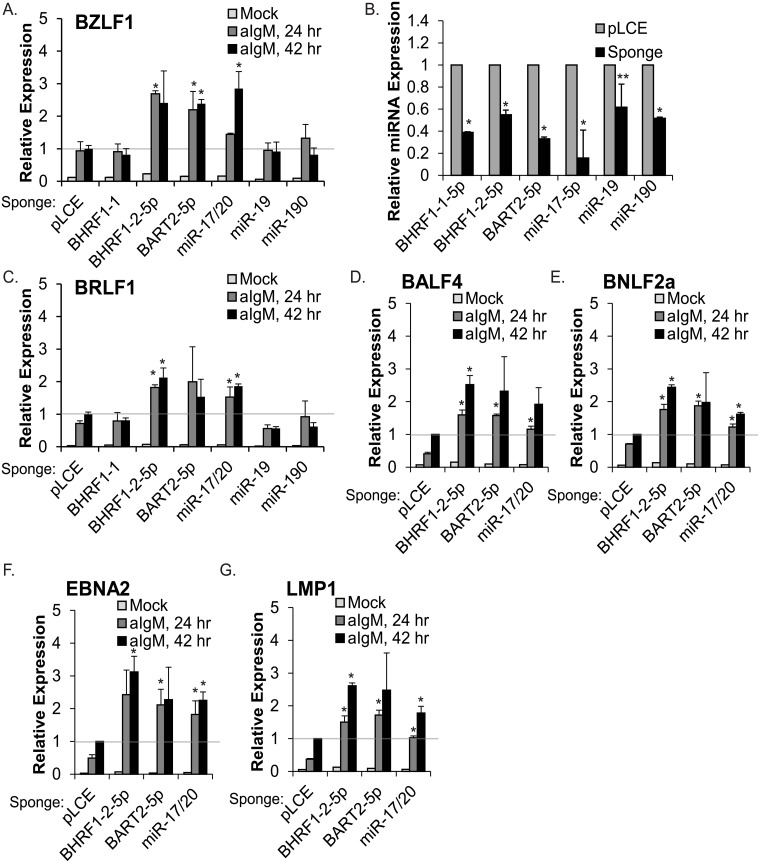
EBV miR-BHRF1-2-5p, miR-BART2-5p, and cellular miR-17 regulate the latent to lytic switch. A and C-G. MutuI cells were transduced with pLCE control vector or sponge inhibitors to indicated EBV or cellular miRNAs, then treated for 24 or 42 hrs with 5 ug/mL anti-IgM. Total RNA was harvested and assayed by qRT-PCR for EBV gene expression as indicated. Reported are the averages of two independent experiments with qPCR performed in duplicate; expression levels are normalized to GAPDH and shown relative to mock treated cells (harvested at 42 hrs) for each miRNA sponge inhibitor. B. miRNA levels in anti-IgM treated, sponged MutuI cells assayed by qRT-PCR. Values are normalized to miR-16 and shown relative to pLCE control cells for each respective sponge inhibitor. Reported are the averages of two independent experiments with qPCR performed in duplicate. *By Student’s t test, p<0.05; **p<0.06. p-values were derived by comparing sponged cells to pLCE (control) cells for each treatment.

We then tested whether EBV miRNAs could directly influence BCR-mediated EBV reactivation from latency, using sponge inhibitors to disrupt the activity of individual miRNAs in Mutu I cells. We focused on two EBV miRNAs: (i) miR-BHRF1-2-5p, for which we were able to confirm multiple targets directly linked to BCR signaling and (ii) miR-BART2-5p, which reduces BCR-mediated NF-kappaB activation (Fig 1) and also targets EBV BALF5 [57]. Additionally, we included inhibitors for miR-BHRF1-1, which was previously reported to enhance virus reactivation by targeting the ubiquitin ligase RNF4 [58], and two cellular miRNAs- namely, the miR-17/20 family that regulates EBV BHRF1 and LMP1 [17,19,32] and miR-190 that was reported to inhibit phorbol ester or BCR-mediated reactivation of EBV+ BL cells [59]. An inhibitor to cellular miR-19 was included as an additional control. Mutu I cells were transduced with pLCE control or the individual miRNA sponge inhibitors, then stimulated with anti-IgM for 24 hr or 42 hr (Fig 7A). We observed ~40–50% reduction in steady-state, mature miRNA levels in the presence of the sponge inhibitors, indicating the sponges were functional and in this experimental context, contributing to miRNA decay (Fig 7B).

To monitor lytic reactivation in MutuI cells, BZLF1 and BRLF1 transcripts were measured by qRT-PCR. Surface Ig cross-linking led to ~8 to 10-fold induction of EBV IE gene expression in pLCE control cells by 24 hr (Fig 7A and 7C). We observed comparable ~10-fold induction of EBV IE transcripts in the presence of miR-BHRF1-1, miR-BART15-3p, or miR-19 inhibitors, as well as the miR-190 inhibitor (contrary to a previous report [59]), demonstrating these miRNAs do not significantly impact the initial stages of EBV reactivation in Mutu I cells. Upon suppression of miR-BHRF1-2-5p, miR-BART2-5p, or miR-17/20 activity, however, we observed significant increases in BZLF1 and BRLF1 levels following anti-IgM treatment compared to control cells (Fig 7A and 7C). To investigate this further and ensure that the full lytic program was initiated, we measured additional early and late viral transcripts (BALF4, BNLF2a, EBNA2, LMP1) in anti-IgM treated cells. Compared to control cells, all four EBV transcripts were significantly upregulated at both 24 hr and 42 hr in cells with inhibitors to miR-BHRF1-2-5p, miR-BART2-5p, or miR-17/20 (Fig 7D–7G). These results demonstrate that miR-BHRF1-2-5p, miR-BART2-5p, and miR-17/20 regulate the amplitude of virus reactivation as triggered through surface Ig cross-linking.

**Fig 8:**
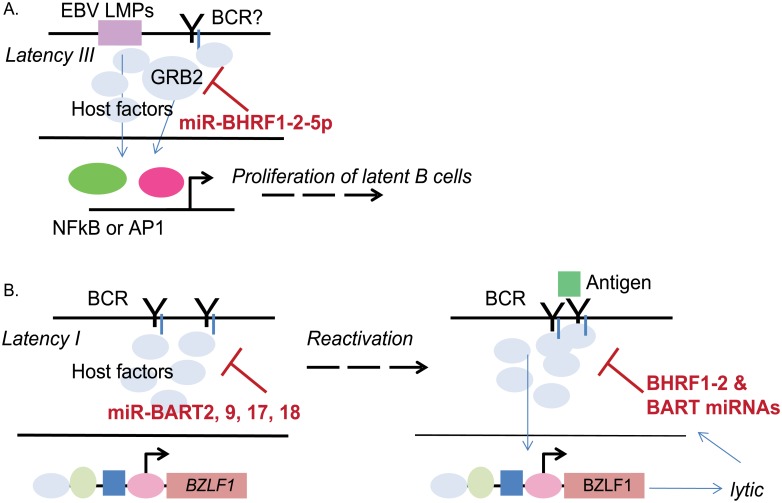
Hypothetical model by which EBV miRNAs, such as miR-BHRF1-2-5p, regulate signal transduction components downstream of BCR and modulate the latent/lytic switch. A. EBV miR-BHRF1-2-5p regulates Grb2 protein levels in multiple EBV-infected B cell types which contributes to B cell proliferation (irrespective of an intact BCR). B. Multiple EBV miRNAs, including miR-BHRF1-2-5p, attenuate signaling through the BCR. Disruption of EBV miR-BHRF1-2-5p and miR-BART2-5p activities, in particular, impact the amplitude of virus reactivation when triggered by antigen cross-linking.

The overall increase in lytic reactivation upon miR-BART2-5p inhibition could be attributed, in part, to the targeting of EBV BALF5 encoding the viral DNA polymerase catalytic subunit [57]. To separate this activity from effects of miR-BART2-5p on the BCR signaling pathway, we also measured lytic reactivation at time points earlier than 24 hr. Increased BZLF1 levels were detectable in miR-BART2-5p sponged cells as early as 5 hours post anti-IgM stimulation, which occurs prior to BALF5 expression and virus replication, suggesting that miR-BART2-5p is indeed hindering reactivation signals mediated through BCR signals (S7 Fig). Intriguingly, we did not see significant changes in basal BZLF1 or BRLF1 expression in mock treated Mutu I cells for any of the miRNA inhibitors (Fig 7A and 7C). These data lead us to conclude that EBV miRNAs such as miR-BHRF1-2-5p and miR-BART2-5p are not actively blocking spontaneous lytic replication, but are positioned to respond to and antagonize extracellular stimuli that lead to virus reactivation (Fig 8).

## Discussion

In this study, we evaluated the role of EBV miRNAs in BCR signaling, which has implications for the proliferation of latently infected B cells and importantly, for influencing virus reactivation from the latent state. Antigen stimulation of the BCR induces multiple intracellular signaling pathways, and latent viral proteins have been previously demonstrated to manipulate BCR signaling components and/or the pathways (i.e. NF-kappaB, AP1, PI3K, MAPK) triggered through BCR activation. As viral miRNAs are non-immunogenic viral gene products that are expressed throughout multiple stages of EBV infection, we hypothesized that the viral miRNAs would be prime candidates to coordinately modulate the signaling cascade initiated through the BCR.

Through functional screens, we demonstrate that a subset of five EBV miRNAs desensitizes BL cells to BCR cross-linking, subsequently attenuating downstream transcriptional activation of NF-kappaB and/or AP1 (Fig 1). By analyzing existing B cell miRNA targetome datasets which globally captured EBV miRNA interactions [17,18,60], we find that signal transducers as well as multiple components of the pathways situated downstream of the BCR can be regulated by the viral miRNAs. 54 host targets were identified as being associated with BCR pathways (Figs 2 and 3), and using biochemical assays, we validated several new interactions between EBV miRNAs and the cellular 3’UTRs (GRB2, PAG1, RAC1, INPP5D, IKBKB, CDKN1A, FOXO3) (Fig 3), thereby providing insight into the underlying molecular mechanisms by which these miRNAs attenuate BCR signaling.

Our experiments highlight that the EBV BHRF1-2 miRNAs, in particular, act as novel and potent regulators of BCR signal transduction and consequently, can restrict entry into the lytic cycle initiated by BCR engagement. The BHRF1-2 miRNAs are evolutionarily conserved, with homologs encoded by multiple lymphocryptoviruses (LCV) that infect Old World non-human primates [32,61]. The high sequence conservation with other LCV BHRF1-2 miRNA homologs supports the notion that these miRNAs are of particular importance to the viral life cycle. To date, there are only a few characterized targets of the EBV BHRF1-2 miRNAs and even fewer targets linked to function [11,17,43,62]. Here, we evaluated several interactions between the BHRF1-2 miRNAs and host 3’UTRs (MALT1, PRDM1, SOS1, PLCG1) that have been examined in other reports in the literature [11,43,44], and further confirmed novel interactions with the GRB2, RAC1, PAG1, and INPP5D 3’UTRs (Fig 3). Site-directed mutagenesis of miRNA seed-match sites in several of these 3’UTRs abrogated luciferase reporter knockdown, clearly demonstrating that the BHRF1-2 miRNAs interact with these target RNAs through the identified binding sites. Commonly, Grb2, Plcg1, Sos1, and Pag1 interface with receptor tyrosine kinases (RTKs) to link the BCR with downstream pathways. The fact that multiple players in BCR responses are inhibited by the BHRF1-2 miRNAs can thus explain the strong disruption of BCR signaling.

Grb2 is a ubiquitously expressed signaling adaptor protein that interacts with numerous growth factor and antigen RTKs via its src-homology domains [40,63]. The role of Grb2 in regulating BCR signaling and normal B cell responses (i.e. not in the context of EBV infection) is not completely understood. Grb2 was initially described as a negative regulator of B cell activation [63], following studies demonstrating that ablation of Grb2 from mature B cells in mice enhanced proliferation responses to BCR cross-linking and altered lymphoid follicle organization in germinal centers [64,65]. Intriguingly, B cell-specific Grb2-/- mice exhibited reduced numbers of splenic B cells in the periphery as well as reduced follicular B cells, and lacked germinal centers in the spleen but not in other secondary lymphoid organs [40,64]. More recent studies demonstrate that Grb2 acts as a positive regulator of B cell activation; re-introduction of the protein into Grb2-deficient splenic murine B cells enhanced BCR-induced calcium mobilization [66]. In reconciling these observations, it is likely that Grb2 has a differential role in B cell activation where cell context and stage of B cell development are critical factors. Studies on FGFR2 (fibroblast growth factor receptor 2) expressing cancer cells demonstrate that Grb2 function is concentration dependent and when interacting with RTKs, focally concentrated Grb2 levels contribute to receptor pre-dimerization in the absence of external stimulation while at the same time prevent uninitiated downstream responses [67]. Of note, Plcg1 has been shown to compete with Grb2 for RTK interactions; in the context of FGFR2 signaling, knocking down Grb2 allows for increased recruitment of Plcg1 to the receptor which has implications for increased metastatic potential [67]. In human BL cells, we observed that shRNA-mediated knockdown of GRB2, but not PLCG1, inhibited BCR-triggered signaling events, thereby phenocopying BHRF1-2 miRNA activity (Fig 4). Thus, in this context, our findings are in line with Grb2 acting as a positive regulator to amplify BCR-induced signals.

Notably, GRB2 as well as SOS1 are regulated by non-coding RNAs in other g-herpesvirus infection systems, suggesting this may be a common strategy employed by g-herpesviruses to manipulate RTK signaling. KSHV miR-K10a, a mimic of cellular miR-142-3p, targets the SOS1 3’UTR [18]. KSHV miR-K12-4-3p and miR-K9-5p target the GRB2 3’UTR and reduce Grb2 protein levels during KSHV infection [18,68]. In contrast, Herpesvirus saimiri (HVS), a non-human primate g-herpesvirus that naturally infects squirrel monkeys and is transforming in marmoset T cells, upregulates Grb2 levels by counteracting the activity of cellular miR-27 [69]. While GRB2 has been shown to be regulated by these other g-herpesviruses [18,68,69], the functional significance of these interactions has not been fully determined. Grb2 is conventionally linked to Ras and MAPK signal transduction pathways through its cytoplasmic binding partners, such as PLCG1 and the Ras guanine nucleotide exchange factor encoded by SOS1. In our experiments, we found that shRNAs knocking down GRB2 or SOS1 in particular phenocopied BHRF1-2 miRNA function and had a negative impact on NF-kB activation in response to BCR cross-linking. While this leads us to conclude that disruption of GRB2 or SOS1 by RNA-interference mechanisms impairs antigen receptor signaling, the fact that NF-kappaB activity was reduced is not so easily explained. Due to the numerous binding partners of Grb2, we speculate one way this might occur is through indirect effects of secondary messengers, such as DAG (diacylglycerol) and IP3 (inositol-1,4,5-triphosphate), that are generated from PIP2 (phosphatiyl-4,5-bisphosphate) catalysis in response to BCR triggering. DAG activates protein kinase C which can subsequently activate NF-kappaB, among other factors. Thus, apart from the canonical Ras and MAPK pathways, impairment of a BCR signal integrator such as Grb2 has multiple apparent consequences for downstream signaling pathways.

Through loss-of-function experiments, our study further reveals that miR-BHRF1-2-5p activity and optimal Grb2 levels are necessary for efficient proliferation of EBV transformed B cells. In concordance with previous reports, LCLs established with BHRF1-2 miRNA mutant viruses exhibited growth defects compared to those established with wild-type EBV B95-8 [45,48] (Fig 5 and S3 Fig). While these previous reports have speculated that inhibition of cellular factors by the BHRF1 miRNAs is necessary to provide a favorable environment for effective B cell transformation and/or growth of LCLs, the cellular targets have been elusive. Work from the Delecluse lab demonstrated that mutation of the region encoding the BHRF1-2 miRNAs affects processing of the adjacent BHRF1-3 primary miRNA; thus, B cells infected with BHRF1-2 miRNA mutant viruses exhibit reduced levels of miR-BHRF1-3 when compared to B cells infected with wt B95-8 [48]. This complicates interpretation of experiments measuring B cell transformation efficiencies at early stage infection when viruses harboring mutations in individual or multiple BHRF1 miRNAs are compared [45,48,49]. By comparing different iterations of BHRF1 miRNA mutant viruses, these prior studies proposed that miR-BHRF1-3 is a vital part of EBV-mediated B cell transformation [48,49]. Our results argue that the conserved BHRF1-2 miRNAs are the primary contributors in maintaining the growth of transformed B cells (Figs 5 and 6, S4H Fig). We tested whether miR-BHRF1-3 could be a contributing factor at later stages by measuring the growth of LCLs generated with BHRF1-2, BHRF1-3, or triple BHRF1 miRNA mutant viruses (S3 Fig). Consistent with previous studies, LCLs lacking only the BHRF1-2 miRNAs or all three BHRF1 miRNAs had dramatically reduced growth capabilities compared to wt virus; however, we did not observe any growth differences with the BHRF1-3 miRNA mutant virus. This discrepancy could be explained by the time points examined; it is possible that growth defects associated with abrogation of miR-BHRF1-3 occur at early stages after de novo infection and may resolve later once LCLs become established.

Recent work from Poling et. al. investigated whether growth deficiencies in LCLs generated with a virus lacking all BHRF1 pre-miRNAs could be reversed [50]. In contrast to our experiments which focused specifically on a mutant lacking only the BHRF1-2 miRNAs, simultaneous expression of all BHRF1 miRNAs in trans failed to rescue the triple mutant. Molecular analysis by Poling et. al. further revealed that mutations in the BHRF1 pre-miRNA stem loops induce altered splicing patterns in the EBNA-LP transcripts, which the authors rationalized were irreversibly detrimental to LCL growth [50]. Of note, EBNA-LP levels are also upregulated in viral mutants where the BHRF1-2 or BHRF1-3 miRNAs are individually inactivated [45]. We found that LCL growth was crippled predominantly when the BHRF1-2 miRNAs were absent, but not the BHRF1-3 miRNAs (Fig 5 and S3 Fig), presenting the likelihood that factors in addition to EBNA-LP are responsible for changes in growth patterns. We used multiple strategies, including shRNAs as well as miR-BHRF1-2-5p sponge inhibitors, to demonstrate that miRNA-mediated control of host targets such as GRB2 indeed provides a growth advantage for latently infected B cells.

Long-lived, memory B cells are thought to be a primary reservoir for persistent EBV infection in vivo as the virus is able to gain access to this compartment through viral gene expression programs that manipulate B cell growth and differentiation [5]. While the stimulus for EBV reactivation in vivo is not fully known, activation of BCR signaling is thought to represent the most physiologically relevant trigger for lytic reactivation in vitro. In examining the functional consequences of perturbing miRNA activity, we uncovered roles for EBV miR-BHRF1-2-5p, miR-BART2-5p, and unexpectedly, miR-17-5p in the latent to lytic switch (Fig 7). Herpesviruses have complex relationships with miR-17 family members. Human cytomegalovirus, for example, induces selective turnover of miR-17 family members via a viral decay element in order to accelerate lytic infection [70]. Our data show that inhibition of miR-17 can also accelerate lytic replication for EBV. In contrast to CMV, EBV and other g-herpesviruses do not appear to have mechanisms for inducing miR-17 turnover, and in fact, seem to require miR-17 or miR-17-like activity during latent infection [71,72]. Furthermore, EBV miRNAs collectively share a significant number of targets with miR-17 [19], arguing that repression of miR-17 target genes is a key part of the latent g-herpesvirus life cycle. Although the mechanism(s) by which miR-17 controls EBV reactivation remain to be elucidated, we note that Ago-CLIP studies have cataloged miR-17 targets such as SOS1, VAV2, and GAB1 [17–19] that indicate the phenotype, like that of the EBV miRNAs, is linked to cellular responses associated with BCR triggering.

By suppressing signals that arise via engagement of the BCR, we propose that EBV miRNAs such as miR-BHRF1-2-5p and miR-BART2-5p protect latent cells from aberrant reactivation (Fig 8). We further postulate that the BHRF1-2 miRNAs play additional roles in dampening lytic reactivation. Cross-linking of the BCR activates latently infected memory B cells to differentiate into plasma cells [23]. Previous studies report that miR-BHRF1-2-3p targets the PRDM1 3’UTR, which we confirmed in this study using luciferase reporter assays [17,43] (Fig 3). Blimp1, encoded by PRDM1, is a master B cell transcription factor required for the differentiation of B cells into plasma cells. Overexpression of Blimp1 in several BL cell lines, particularly Wp-restricted BL, is sufficient to induce lytic reactivation, which is thought to occur through Blimp1-mediated activation of the IE promoters driving BZLF1 (Zp) and BRLF1 expression [24]. While it remains to be tested, miR-BHRF1-2-3p could potentially suppress lytic reactivation by repressing Blimp1. It is conceivable that 5p miRNA, miR-BHRF1-2-5p, also attenuates EBV lytic replication by regulating transcription factors involved in B cell differentiation. SP1 (specificity protein 1), for example, is a cellular transcription factor that also binds to and activates Zp [73], and a binding site for miR-BHRF1-2-5p was previously reported in the SP1 3’UTR [17]. Given that we did not observe spontaneous lytic reactivation upon miR-BHRF1-2-5p inhibition in LCLs, DLBCLs, or BL (Figs 6 and 7), we speculate that cellular context as well as cues from the extracellular environment are major participants in miRNA-influenced decisions impacting virus reactivation.

An intriguing question that remains is how miR-BHRF1-2-5p physically exerts function in latency I BL cells during induction of the lytic cycle. Unlike miR-BART2-5p and cellular miR-17, the BHRF1-2 miRNAs are not detectable in latency I until cells respond to reactivation cues (S6B Fig); yet, introduction of an inhibitor against miR-BHRF1-2-5p enhances lytic gene expression (Fig 7). One possible explanation is that within this heterogeneous population, the response of individual cells to reactivation stimuli is asynchronous. In this scenario, some cells will conceivably respond more quickly and express the BHRF1-2 miRNAs before other cells become responsive. Previous reports have demonstrated that the EBV BHRF1 miRNAs can be transferred between EBV+ B cells and uninfected T cells in co-culture experiments [74]. Moreover, delivery of functional EBV miRNAs via exosomes has been demonstrated by several groups [75,76]. While further investigation will be required, we speculate that the BHRF1-2 miRNAs may be transferred to neighboring cells to exert their effects, thereby controlling the overall level of virus reactivation in the cell pool.

In summary, we have shown that a subset of EBV miRNAs attenuates signaling through the BCR and governs aspects of EBV reactivation. An important goal for future studies will be to examine specific contributions from additional viral miRNAs as well as cellular miRNAs that are hijacked by the virus in order to more fully decipher the extent to which post-transcriptional regulatory mechanisms facilitate latent/lytic cell state transitions. Gaining a clear understanding of the mechanisms regulating latency and entry into the lytic replication cycle is a critical part of designing effective treatments for viral disease. Our findings thus provide valuable insight into how viral miRNAs functionally contribute to viral latency and have utility in aiding development of miRNA-focused therapeutic strategies.

## Materials and methods

### Cell culture, lentivirus, and transductions

B cell lines (BJAB, LCLs, DLBCLs, MutuI) were maintained at 37°C in a 5% CO_2_-humidified atmosphere in RPMI-1640 supplemented with 15% fetal bovine serum (FBS) (unless otherwise stated) and 1% penicillin, streptomycin, and L-glutamine (P/S/G). MutuI BL cells exhibit cell surface IgM (uK+) [55] and were kindly provided by Dr. Erik Flemington at Tulane University. BJAB (EBV-neg germinal-center derived BL cell line), EBV wild-type, EBV B95-8 and BHRF1-2 miRNA mutant LCLs (IBL-LCLd3, SDLCL, LCL35, LCLBACWT, LCLBACD2) originated from the laboratories of Dr. Bryan Cullen or Dr. Micah Luftig at Duke University and are previously described in [17,32,60]. Additional LCLs were generated with EBV B95-8 2089 or EBV BHRF1 miRNA mutant viruses, with kind permission from Dr. H.J. DeLecluse at the German Cancer Research Centre, using a multiplicity of infection = 2 Raji-GFP units [11,45]; LCL-WT, LCL-D2, LCLWT-16.1, and LCLD2-16.1 are previously described in [11]. DLBCL cell lines (IBL1, BCKN1, IBL4) are previously described [53] and were kindly provided by Dr. Ethel Cesarman at Weill Medical College of Cornell University. Human primary B lymphocytes (PBMCs) used in this study were isolated from anonymous whole blood purchased from Research Blood Components.

HEK293T cells (originating from Dr. Bryan Cullen’s laboratory) were maintained in high glucose DMEM supplemented with 10% FBS and 1% P/S/G. For preparation of lentiviruses, HEK293T cells were plated in 15-cm plates in complete media and transfected using Polyethylenimine (PEI) with 7.5 ug pL-based lentivector, 4.5 ug pDeltaR8.75 and 3 ug pMD2G. Media was changed to complete RPMI-1640 between 8 hrs and 16 hrs post-transfection. Lentiviral particles were harvested by sterile filtration of the supernatant using a 0.45 micron filter at 48 and 96 hrs post-transfection and used to transduce ~1 to 5 x 10^6 cells. BJAB, MutuI, LCLs, and DLBCLs were transduced with one (BJAB) or two (LCLs, DLBCLs, and MutuI) rounds of lentivirus and monitored by fluorescent microscopy for transduction efficiency by green fluorescent protein (GFP) and/or mCherry expression. DLBCLs were further subjected to sorting by flow cytometry to obtain >95% pure populations of GFP-positive cells. To induce lytic reactivation, MutuI cells were spun down and plated at 1 x 10^6 cells in fresh media containing soluble anti-IgM (Sigma) at concentrations and times indicated in figure legends (2.5–5 ug/mL for 22–48 hrs).

### Plasmids

EBV miRNA expression vectors in pLCE are previously described [16,17,32]. pLSG-PRDM1 is previously described [18] and was generously provided by Dr. Eva Gottwein at Northwestern University. psiCheck2-RAC1 and psiCheck2-IKBKB are previously described [77,78]; psiCheck2-FOXO3.2 (containing the second half of the FOXO3 3’UTR) was generously provided by Dr. Jay Nelson’s laboratory at Oregon Health and Science University. To generate other 3’UTR luciferase reporters, 3’UTRs were PCR amplified from genomic DNA of EBV-infected B cells and cloned into the XhoI and NotI sites downstream of *Renilla* luciferase in the psiCheck2 dual luciferase reporter vector. When achievable, the entire 3’UTR was cloned for a given target. For longer 3’UTRs, a minimum of 1 kb containing the region predicted to be targeted by each miRNA was cloned. Mutant 3’UTR reporters, containing nucleotide changes in miRNA seed match sites as identified by PAR-CLIP, were generated by Phusion Taq site-directed mutagenesis.

Lentiviral vector miRNA sponge inhibitors contain six to eight imperfect, tandem decoy binding sites for a single miRNA as previously described [11,17,79]. Sponge oligonucleotides contain flanking KflI sites and were annealed and concatamerized prior to cloning into the GFP 3’UTR of pLCE. The control sponge (pLCE-CXCR4s) is previously described [17,79]. miR-30-based shRNA constructs were cloned into the XhoI/EcoRI sites of pL-CMV-mCherry vector using GRB2.3140, MALT1.3197, RAC1.424, INPP5D.1191 oligonucleotides as described in [80]. shRNA constructs for PLCG1 and SOS1 are previously described [11].

### MTT assays

Cells were maintained in log phase and split 1:2 or 1:3 one day prior to seeding into 96-well plates at 2.5 x 10^3 cells/well in complete RPMI media without phenol red. At each time point, 100 ul per well of MTT (3-(4,5-dimethylthiazol-2-yl)-2,5-diphenyltetrazoliumbromide) reagent (5 mg/ml in phosphate-buffered saline) was added. Cells were incubated for 2 hrs at 37°C, lysed in MTT solvent (isopropanol containing 0.5% NP40 and 4 mM HCl), and incubated for an additional 2 hrs at 37°C. Lysates were read using an ELISA plate reader (Absorbance = 562 nm), and relative cell growth was determined by comparing absorbance values of cell lysates at time zero (T0) to the values at 24, 48, or 72 hrs post-plating (Tn) as indicated.

### Growth curves

Growth curves were performed 4 to 7 days after final transductions when LCLs were >75% GFP positive by microscopy and in the log-phase of growth. FAC-sorted DLBCLs were rested for 2 days after sorting and prior to plating for growth curves, To assay growth, cells were split 1:2 in BJAB-conditioned complete media one day prior to plating and plated in triplicate or quadruplicate at 2 x 10^5 cells per mL in 24-well plates. Viable cells were counted by trypan blue exclusion using a hemacytometer at time points indicated.

### Western blotting

Cells were lysed in NP40-lysis buffer (50 mM HEPES pH 7.5, 150 mM KCl, 2 mM EDTA, 1 mM NaF, 0.5% (vol/vol) NP40, 0.5 mM dithiothreitol (DTT)) and protein concentrations determined using the BCA protein assay kit (Thermo Scientific). 20 ug of total protein per lane was resolved on 10% Tris-glycine SDS-PAGE and transferred onto Immobilon PVDF membranes. Following blocking in 5% milk in TBS-Tween, blots were probed with primary antibodies to Grb2 (#3972, Cell Signaling Technology), Malt1 (B-12, sc-46677, Santa Cruz), Plcg1 (D9H10, #5690, Cell Signaling Technology), beta-Actin (clone C4, sc-47778, Santa Cruz), or Gapdh (#ab8245, Abcam), then probed with horse-radish peroxidase (HRP) conjugated secondary antibodies (anti-rabbit IgG or anti-mouse IgG). Blots were developed using enhanced chemiluminescent substrate (Pierce) and exposed to film. Protein levels were quantified from scanned films using NIH ImageJ.

### Luciferase reporter assays

NF-kappaB and AP1 activity was assayed using BJAB luciferase reporter cell lines. BJAB-NFkBLuc cells are previously described [11]. To generate BJAB-NFkB-GL4.32 or BJAB-AP1-GL4.44, 3 ug linearized plasmid (pGL4.32 or pGL4.44 (Promega)) was transfected into BJAB cells using the Amaxa Nucleofector II device (Lonza), program G-016, in 100 ul of Ingenio electroporation solution (Mirus). Cells were placed under hygromycin selection for two weeks. EBV miRNAs or shRNAs were subsequently introduced by lentiviral transduction. For NF-kappaB activation, 1 x 10^5 cells per well were plated in 96-well black-well plates, stimulated for 18 hrs with 5 or 10 ug/ml anti-IgM (Sigma) as indicated, and then lysed in 1X passive lysis buffer (Promega). Luciferase activity was assayed using the Dual Luciferase Reporter Assay System (Promega) and a Veritas microplate luminometer (Turner Biosystems) with dual injectors. For AP1 activation, cells were stimulated for 6 hrs with 10 ug/ml anti-IgM (Sigma), and lysed in 1X passive lysis buffer (Promega). AP1 firefly luciferase activity is normalized to total protein levels quantified with the BCA Protein Assay kit (Pierce).

### 3’UTR reporter assays

HEK293T cells plated in 96-well black-well plates were co-transfected with 20 ng of 3’UTR reporter and 250 ng of control vector (pLCE) or EBV miRNA expression vector [16,17,32] using Lipofectamine2000 (Thermofisher) according to the manufacturer’s instructions. 48–72 hrs post-transfection, cells were collected in 1X passive lysis buffer (Promega). Lysates were assayed for luciferase activity using the Dual Luciferase Reporter Assay System (Promega) and a luminometer with dual injectors. All values are reported as relative light units (RLU) relative to luciferase internal control and normalized to pLCE control vector.

### Quantitative RT-PCR (reverse transcription-polymerase chain reaction) analysis

Total RNA was extracted using TRIzol (Thermofisher). For detection of cellular or EBV transcripts, RNA was DNAse-treated and reversed transcribed using MultiScribe (Thermofisher) with random hexamers. Transcripts were detected using SYBR Green qPCR and oligonucleotides designed to amplify gene specific regions of ~200 bp. Primers for amplification of EBV transcripts BZLF1, BRLF1, BALF4, BNLF2a, and BHRF1 are described in [81]. Oligonucleotides are listed in Table S1. miRNAs were detected using commercial Taqman assays or miRNA stem-loop qRT-PCR assays as previously described [11,49]. miRNA levels are reported relative to miR-16 (assay #000391, Thermofisher) or U6 (assay #001973, Thermofisher) as indicated. All PCR reactions were performed in technical replicates (duplicates or triplicates).

### PARCLIP datasets and pathway analysis

PAR-CLIP datasets for EBV+ BC1 cells and EBV B95-8 or wild-type LCLs are previously described [17,18,32,60]. Raw fastq files were preprocessed as described previously and reads ≥13 nt were aligned to the human genome (HG19) and either the EBV B95-8 genome (V01555.2) or the wild-type EBV1 genome (NC_007605.1) using Bowtie (-v 3 –m 10) [17,18,82]. Mapped reads were analyzed by PARalyzer [83] to define 3’UTR interaction sites for EBV BART2, 9, 17, 18, and BHRF1-2 miRNAs. A comprehensive list of cellular genes associated with BCR signaling was curated from six publicly available databases (Reactome, Panther Pathways, NDeX, KEGG, PathCard, and NetPath), and gene identifiers of the EBV miRNA 3’UTR interactions were compared to determine targets related to BCR signaling. Pathways interactions were compiled and drawn in PathVisio [84,85].

### Statistical analyses

All luciferase and PCR data are reported as mean with standard deviations (S.D.) and values are derived from at least three independent experiments, unless otherwise stated, with technical replicates. Statistical significance was determined by paired Student’s t test, performed using Microsoft Excel 2010, and values p < 0.05 were considered significant.

## Supporting information

S1 Fig EBV BHRF1-2 expression does not significantly alter steady-state levels of target RNAs related. A. and B. Western blot analysis of Malt1 or Grb2 levels in LCLs. Established LCLs are infected with EBV B95-8 2089 (WT) or BHRF1-2 miRNA mutant virus (D2). C-E. qRT-PCR analysis of EBV BHRF1-2 targets. RNA was isolated from established LCLs infected with wild-type EBV B95-8 2089 or BHRF1-2 miRNA mutant EBV (Donors 1–3) (n = 2). F-H. RNA was isolated from LCLBACD2 (n = 2), LCL-D2 (n = 3), or BJAB (n = 2) cells stably expressing GFP (pLCE) or the BHRF1-2 miRNAs. In C-H, cellular transcript levels were assayed by qRT-PCR. Values are normalized to GAPDH and reported relative to control cells (pLCE). Average expression values and standard deviations were calculated from two or three independent experiments. I. EBV miRNA expression in 293T cells (corresponds to miRNAs tested in Fig 3). RNA was harvested from 293T cells transfected with control vector (pLCE) or individual EBV miRNA expression vectors (pLCE-miR) at 48 hrs and from Mutu I cells treated with 5 ug/mL anti-IgM for 48 hrs. miRNAs were detected by qRT-PCR. Values are normalized to cellular miR-16 and reported relative to levels in Mutu I cells. Average expression values and standard deviations were calculated from two experiments.
(EPS)

S2 Fig Validation of shRNAs. A. shRNAs stably expressed in BJAB cells reduce target RNA levels. BJAB cells were stably transduced with mCherry or mCherry-shRNA expressing lentiviruses. RNA was isolated and cellular transcripts were assayed by qRT-PCR. Values are normalized to GAPDH and reported relative to control cells (pLmCherry). Average expression values and S.D. were calculated from two independent experiments. B. shRNA knockdown of target genes in LCL-D2 (see Fig 5). RNA was harvested from LCL-D2 cells 7-10d post transduction with mCherry or the individual shRNAs (corresponds to Fig 5B and 5C). Levels of target genes were assayed in duplicate by qRT-PCR analysis. Expression levels are normalized to GAPDH and reported relative to control (mCherry) cells.
(EPS)

S3 Fig BHRF1-2 miRNAs contribute to the growth of established LCLs. A. Growth curves of established LCLs at > 8 weeks post-infection. LCLs (derived from same donor) were generated with either wild-type (LCL-WT) or BHRF1-2 miRNA mutant (LCL-D2) EBV and maintained in log-phase in complete media containing 15% FBS. B and C. Proliferation of wild-type or BHRF1-2 miRNA mutant LCLs as determined by MTT assay (Donor 2 = LCL-WT or LCL-D2; Donor 4 = LCL17.1-WT, -D2,-D3 or -D123 (mutated for BHRF1-2, -3, or all BHRF1 miRNAs)). A = Absorbance at 562 nm, T = time, n = 24, 72, or 96 hr as indicated. Values at Tn are normalized to the absorbance values at 0 hr (A-T0). D-F. Growth curves of LCL-D2, LCLBACD2, or BJAB transduced with control vector (pLCE) or the BHRF1-2 miRNA-expression vector (BHRF1-2). LCLs were split one day prior to initiating growth curves and plated in media containing 10% or 20% FBS as indicated. BJAB cells were grown in media containing 10% FBS. Cell counts were determined at days indicated using trypan-blue exclusion. For D-F., error bars represent S.D. of two to four experiments.
(EPS)

S4 Fig Regulation of GRB2 by miR-BHRF1-2-5p contributes to LCL growth. A-C. Growth curves of EBV B95-8 (SDLCL and LCL35) and wild-type (IBL-LCL3) LCLs following sponge inhibition of miR-BHRF1-2-5p. Cells in log phase were plated in BJAB-conditioned media mixed 1:1 with fresh RPMI-1640 containing 15% FBS and viable cell counts were determined at times indicated by trypan-blue exclusion. Cell growth rates (k values) were calculated between 2 and 5 days post-plating using the following equation: ln(N1/N1) = k(t1-t2), where t = time and N = cell number. Experiments were performed in quadruplicate. D. and E. Control (pLCE-CXCR4s) and sponged (pLCE-BHRF1-2-5ps) SDLCL cells were transduced with mCherry or indicated shRNAs. Cell growth was determined by MTT assay. A = Absorbance at 562 nm, T = time, n = 24, 48, or 72 hr as indicated. Values at Tn are normalized to the absorbance values at 0 hr (A-T0). For D. n = 12 wells and for E., n = 14 wells. *p < 0.05 by Student’s t-test. F. EBV miR-BHRF1-2-5p levels in SDLCL cells expressing miR-BHRF1-2-5p sponge and shGRB2 compared to control cells. Levels were determined by Taqman qRT-PCR and values are relative to cellular miR-16. G. GRB2 expression in SDLCL cells expressing miR-BHRF1-2-5p sponge and shGRB2. Expression levels are normalized to GAPDH and reported relative to control (mCherry) cells. H. Sponge inhibition of miR-BHRF1-1-5p or miR-BART2-5p does not significantly impact LCL proliferation. Growth of control (pLCE-CXCR4s) and sponged SDLCL cells was determined by MTT assay; values at Tn (48 hr) are normalized to absorbance values at 0 hr (A-T0). n = 8 wells. I. EBV miRNA levels in sponged SDLCL cells from (H.). Levels were determined by Taqman qRT-PCR and values are relative to cellular U6. qPCR was run in duplicate.
(EPS)

S5 Fig Viral miRNA expression and viral gene expression in EBV-infected B cell lines. Total RNA was isolated from LCLs, DLBCLs (BCKN1, IBL1, IBL4), and BL (MutuI,Raji) cell lines and assayed for viral gene expression. In A. and B., miRNA levels were determined by Taqman qRT-PCR (performed in duplicate). Values are normalized to cellular miR-16 and reported relative to MutuI cells. For C-E., levels of target viral genes were assayed in duplicate by qRT-PCR analysis. Expression levels are normalized to GAPDH and reported relative to EBV B95-8 SDLCL cells.
(EPS)

S6 Fig Gene expression analysis in reactivated Mutu I cells. A. Detection of EBV lytic transcripts in reactivated MutuI cells. Cells were treated with 2.5 ug/mL anti-IgM for 22 hrs. Total RNA was collected and expression of EBV immediate-early (BZLF1, BRLF1), early (BHRF1, BNLF2a), and late (BALF4) genes was assayed by qRT-PCR. Values are normalized to GAPDH and reported relative to mock treated cells. Averages and S.D. are from two independent experiments; qPCR was performed in duplicate. B. and C. Endogenous EBV BHRF1 or BART miRNA expression in MutuI cells treated with 2.5 ug/mL anti-IgM for 22 hrs. Values are normalized to cellular miR-16 and reported relative to mock treated cells. Averages and S.D. are from two independent experiments; qPCR was performed in duplicate.
(EPS)

S7 Fig Inhibition of miR-BART2-5p activity enhances lytic reactivation as early as five hours post-BCR cross-linking. A. BZLF1 is upregulated in Mutu I cells within 5 hrs after anti-IgM treatment. Mutu I cells were treated with 5 ug/mL anti-IgM for 5 or 48 hrs. Total RNA was harvested and levels of BZLF1, BRLF1, and GAPDH were assayed in duplicate by qRT-PCR. Values are normalized to GAPDH and reported relative to 5 hr anti-IgM treatment. B. Mutu I cells transduced with pLCE or sponge inhibitors for miR-BART2-5p or miR-BHRF1-1-5p were treated with 4 ug/mL anti-IgM for 5 hrs. Total RNA was harvested and assayed for BZLF1 by qRT-PCR. Values are normalized to GAPDH and reported relative to pLCE, anti-IgM treatment. C. miR-BART2-5p expression in anti-IgM treated, BART2-5p-sponged cells from (B.). miRNA levels were determined by qRT-PCR; values are normalized to cellular miR-16 and reported related to anti-IgM treated, pLCE control cells in (B.). PCR was performed in duplicate.
(EPS)

S1 Table Oligonucleotides used for this study. (XLSX)
